# A PRoliferation-Inducing Ligand (APRIL) in the Pathogenesis of Immunoglobulin A Nephropathy: A Review of the Evidence

**DOI:** 10.3390/jcm12216927

**Published:** 2023-11-04

**Authors:** Mohit Mathur, Tak Mao Chan, Kook-Hwan Oh, Laura Kooienga, Min Zhuo, Cibele S. Pinto, Bobby Chacko

**Affiliations:** 1 Visterra, Inc., Waltham, MA 02451, USA; mzhuo@visterrainc.com; 2 Department of Medicine, The University of Hong Kong, Pokfulam, Hong Kong, China; dtmchan@hku.hk; 3 Department of Internal Medicine, Seoul National University Hospital, Seoul 03080, Republic of Korea; khoh@snu.ac.kr; 4 Colorado Kidney and Vascular Care, Denver, CO 80012, USA; lkooienga@cokidneycare.com; 5 Division of Renal Medicine, Department of Medicine Brigham and Women’s Hospital, Harvard Medical School, Boston, MA 02115, USA; 6 Otsuka Pharmaceutical Development & Commercialization, Princeton, NJ 08540, USA; cibele.pinto@otsuka-us.com; 7 Nephrology and Transplantation Unit, John Hunter Hospital, Newcastle, NSW 2305, Australia; bobby.chacko@health.nsw.gov.au; 8 School of Medicine and Public Health, University of Newcastle, Callaghan, NSW 2308, Australia

**Keywords:** APRIL, A PRoliferation-Inducing Ligand, B cells, BCMA, BAFF, IgA nephropathy, TACI, TNFSF13

## Abstract

A PRoliferation-Inducing Ligand (APRIL), the thirteenth member of the tumor necrosis factor superfamily, plays a key role in the regulation of activated B cells, the survival of long-lived plasma cells, and immunoglobulin (Ig) isotype class switching. Several lines of evidence have implicated APRIL in the pathogenesis of IgA nephropathy (IgAN). Globally, IgAN is the most common primary glomerulonephritis, and it can progress to end-stage kidney disease; yet, disease-modifying treatments for this condition have historically been lacking. The preliminary data in ongoing clinical trials indicate that APRIL inhibition can reduce proteinuria and slow the rate of kidney disease progression by acting at an upstream level in IgAN pathogenesis. In this review, we examine what is known about the physiologic roles of APRIL and evaluate the experimental and epidemiological evidence describing how these normal biologic processes are thought to be subverted in IgAN. The weight of the preclinical, clinical, and genetic data supporting a key role for APRIL in IgAN has galvanized pharmacologic research, and several anti-APRIL drug candidates have now entered clinical development for IgAN. Herein, we present an overview of the clinical results to date. Finally, we explore where more research and evidence are needed to transform potential therapies into clinical benefits for patients with IgAN.

## 1. Introduction

The thirteenth member of the tumor necrosis factor superfamily (TNFSF), designated A PRoliferation-Inducing Ligand (APRIL), was first described in 1998 as a molecule with the ability to stimulate tumor cell growth [1]. It is closely related to the B-cell growth factor BAFF (B-cell activating factor of the tumor necrosis factor [TNF] family), with APRIL and BAFF sharing ~30% sequence homology within the TNF domain [2]. Both APRIL and BAFF are able to bind to the TNF receptor transmembrane activator calcium modulator and cyclophilin ligand (CAML) interactor (TACI) and B-cell maturation antigen (BCMA) [3]; however, BAFF differs from APRIL in its ability to bind to a specific receptor, BAFF-R [4]. 

Despite the similarities observed between APRIL and BAFF, the two cytokines work via different molecular pathways and have differing physiologic roles. A genome-wide association study (GWAS) identified APRIL as a key susceptibility locus for IgA nephropathy (IgAN) [5], which is a chronic and progressive condition resulting from abnormal O-glycosylation of IgA1 and the deposition of immune complexes in the kidneys, leading to inflammation and damage [6]. APRIL has also been implicated in several other autoimmune conditions, including systemic lupus erythematosus (SLE), rheumatoid arthritis, alopecia areata, myasthenia gravis, Sjögren’s syndrome, and bullous pemphigoid [7]. APRIL and BAFF are both involved in immunoglobulin (Ig) class switching in B cells, thereby contributing to the pathogenesis of disorders with aberrant Ig production [8]. In addition, APRIL, BAFF, and their receptors are reported to be expressed at high levels in various cancers and appear to be associated with disease severity and treatment response [4].

The functional heterogeneity between APRIL and BAFF in both normal and pathological processes makes them independent candidates for therapeutic intervention. While the role of BAFF in B-cell maturation has made it an obvious target for disease interventions [9,10], research has also begun to elucidate the importance of APRIL, particularly in autoimmune diseases [7]. Specifically, a large body of research has served to corroborate the association between APRIL and IgAN [11,12], paving the way for the development of novel therapies for this potentially life-threatening condition.

## 2. The Biological Roles of APRIL in Health and Disease

### 2.1. APRIL Production

The gene encoding APRIL is located on chromosome 17p13, and the resultant protein is predominantly produced by myeloid cells (monocytes, macrophages, and dendritic cells) and T cells [13]. Normal APRIL expression is induced during bone marrow hematopoiesis [14], but it can also be stimulated in the epithelial cells of the gut, tonsils, and skin [15]. APRIL is synthesized as a type II transmembrane protein and requires intracellular cleavage and processing within the Golgi apparatus, before being secreted in its biologically active form [16].

### 2.2. Physiologic Functions

#### 2.2.1. B-Cell Survival

**Figure 1:**
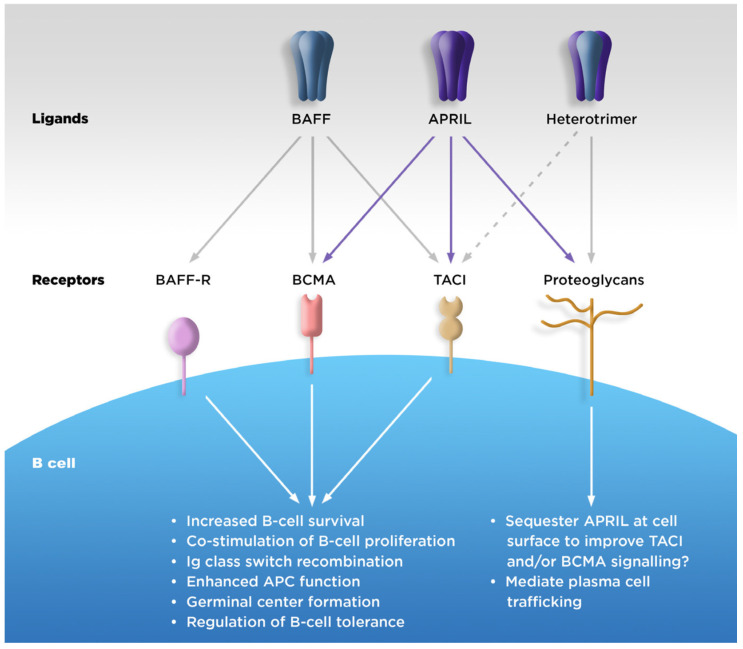
Physiologic roles of APRIL. APC, antigen-presenting cell; APRIL, A PRoliferation-Inducing Ligand; BAFF, B-cell activating factor of the tumor necrosis factor family; BAFF-R, BAFF receptor; BCMA, B-cell maturation antigen; TACI, transmembrane activator calcium modulator and cyclophilin ligand interactor. Figure 1 was adapted from Dillon SR et al. An APRIL to remember: novel TNF ligands as therapeutic targets. *Nat Rev Drug Discov*. **2006**, *5*, 235–246, with permission from Springer Nature (https://www.springernature.com/gp accessed on 10 October 2023).

APRIL, BAFF, and their receptors are known to have specific functions in the process of B-cell maturation and survival (Figure 1). Each step of B-cell maturation within the bone marrow is dependent on APRIL or BAFF, including the development into functional but immature cells, migration to the spleen, antigen encounter, and differentiation into either antibody-secreting cells (ASCs) or antigen-presenting cells (APCs) [17,18]. 

APRIL binds strongly to BCMA and with lower affinity to TACI, while soluble BAFF binds strongly to TACI and BAFF-R, but with weak affinity to BCMA [17]. In addition, APRIL, but not BAFF, is able to bind to cell-surface proteoglycans, which may increase localized APRIL concentration and signaling [17]. Both APRIL and BAFF stimulate B-cell proliferation, but while BAFF is needed for the development of mature B cells, APRIL is involved in the plasma cell survival in the bone marrow [19]. The two cytokines also have independent and non-overlapping roles in Ig isotype class switching in B cells [20,21]. (Table 1).

The binding of BAFF to BAFF-R is critical for mature B-cell development, as demonstrated by the fact that mice deficient in active BAFF have impaired differentiation and a deficit in mature B cells, resulting in impaired humoral responses to antigens [24]. In contrast, primary B-cell maturation in the spleen is not impaired in mice over- or under-expressing APRIL [25,26], but APRIL is necessary to regulate activated B cells and plays a key role in the survival of long-lived plasma cells in bone marrow [19,27].

#### 2.2.2. Ig Class Switching

Another role for APRIL and BAFF is in Ig isotype class switching in B cells [20,21]. The two proteins have non-overlapping roles in this process, whereby class switching can be independently induced via either APRIL–TACI or BAFF–BAFF-R/BAFF–TACI binding [28]. APRIL–TACI and APRIL–proteoglycan binding also contribute to IgA production modulation by B cells and antibody responses to T-cell-dependent antigens [29].

As a B-cell survival factor with the ability to induce T-cell-independent Ig class switching, APRIL plays a key role in the maintenance of the mucosal immunologic barrier [30]. Intestinal epithelial cells produce APRIL and APRIL-inducing cytokines after sensing bacteria through Toll-like receptors. In human IgA1-expressing B cells originating from Peyer’s patches, APRIL triggers sequential class switching to IgA2. This switch enriches the distal intestinal tract with IgA2, which is more resistant to bacterial degradation than IgA1 [31].

APRIL plays a key role in modulating the gut mucosal immune axis, and the hypothesis that the dysfunction of this axis may play a key role in the pathogenesis of IgAN is discussed in more detail in Section 4.2.

#### 2.2.3. Downstream Effects on T Cells

APRIL does not directly contribute to T-cell activation, but its downstream signaling pathways have been implicated in the activation of CD4+ cells [32]. Furthermore, one research group has demonstrated that APRIL knockout mice have a greater number of effector memory T cells, enlarged germinal centers, and elevated IgG responses to T-dependent antigens, despite having normal T- and B-cell development [33].

### 2.3. Association with Disease

Unsurprisingly, given their key roles in B-cell homeostasis and survival, both APRIL and BAFF have also been implicated in the development or maintenance of a myriad of diseases [34]. Aberrant expression levels of APRIL and BAFF have been reported in association with various pathologies, including cancer [4], immunodeficiency [35,36] or autoimmune [37,38] diseases, infection [39,40], and allergies [41]. 

The disruption of B-cell tolerance may be one potential mechanism underlying the role of APRIL in pathologic diseases, particularly immune-related conditions. The plasmablasts and fully differentiated plasma cells responsible for producing autoantibodies are likely to be regulated by APRIL signaling, and APRIL expression has been reported to be associated with the severity and progression of several autoimmune diseases [7,42]. 

In patients with SLE, the aberrant production of APRIL by B cells has been reported [43], in addition to the normal cellular expression by myeloid cells. Furthermore, this uncharacteristic APRIL production could be reproduced by exposing healthy cells to toll-like receptor ligands, which could potentially indicate a B-cell autocrine pathway leading to autoantibody production [44]. Seropositive patients with rheumatoid arthritis have also been reported to have high serum concentrations of APRIL, which correlate with disease activity [45]. Localized APRIL upregulation in the synovial fluid of patients [45] may be a result of the production by local myeloid cells and synovial fibroblasts [46]. Reports have linked increased APRIL serum concentrations with a multitude of other autoimmune diseases [7], and there are also several strands of evidence, including clinical reports, genomic data, and the results from animal models; these have connected alterations in APRIL expression and signaling with the development and severity of IgAN, as detailed below.

## 3. Overview of IgAN

IgAN is considered to be the most common primary glomerular disease worldwide, with estimates placing the overall global incidence at ≥2.5 per 100,000 individuals [47]. IgAN incidence varies by race, ethnicity, and geographic region; Asian populations appear to have a greater risk of developing IgAN compared with Caucasian populations [48,49,50], with up to 60% of biopsy-diagnosed glomerular disease in Asian countries attributed to IgAN [51]. In contrast, the proportion in Europe is around 30%, and in the US, it is about 10% [51]. There is also considerable heterogeneity reported in the clinical manifestations and outcomes between individuals of different races and ethnicities [52,53]. Diagnostic confirmation of IgAN requires a kidney biopsy [54], and once diagnosed, patients could progress to kidney failure within 15 years [55].

**Figure 2:**
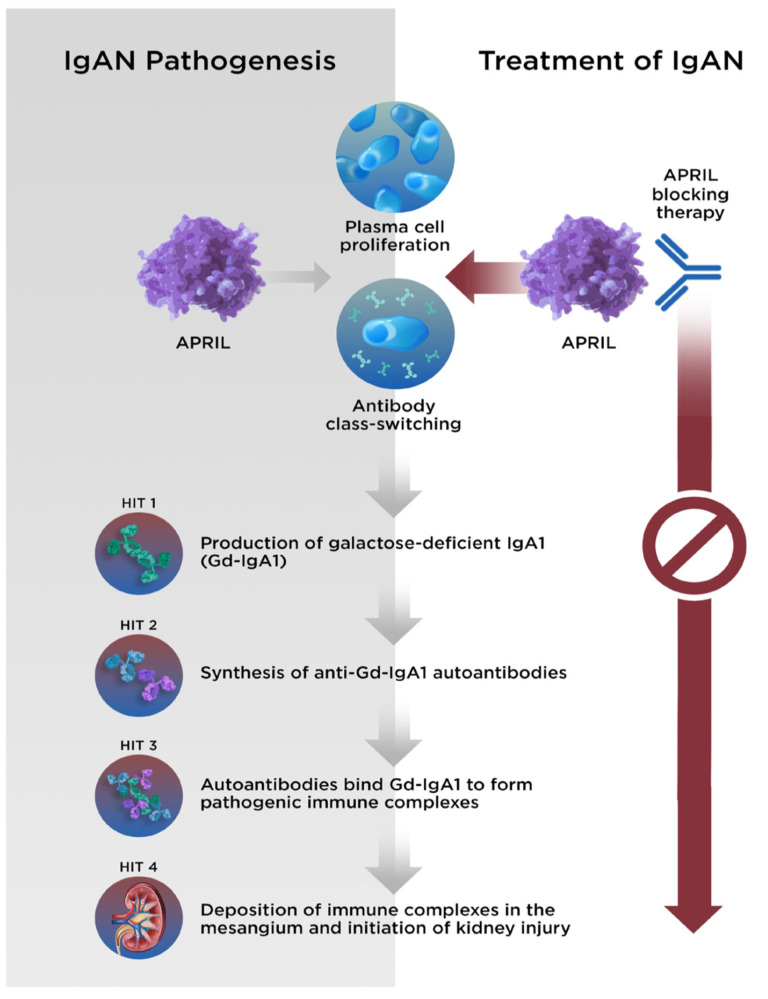
The 4-hit hypothesis describes the pathogenesis of IgAN. With APRIL-blocking therapy, each stage of disease can be halted. APRIL, A PRoliferation-Inducing Ligand; Gd-IgA1, galactose-deficient immunoglobulin A1; IgA1, immunoglobulin A1; IgAN, immunoglobulin A nephropathy.

Although aspects of the underlying pathophysiology remain unclear, the development of IgAN can be described by a 4-hit hypothesis (Figure 2). Hit 1 is an increase in the production and circulation of galactose-deficient IgA1 (Gd-IgA1), which results in the production of autoantibodies (hit 2) against Gd-IgA1. Immune complexes consisting of Gd-IgA1 and autoantibodies then form and circulate (hit 3) and are deposited within the glomerular mesangium (hit 4) [56,57]. Subsequently, cytokine production at the sites of the immune complex deposition result in localized inflammation and activation of the complement system, renin angiotensin system, and stimulation of mesangial proliferation [58].

IgA antibodies provide the first line of defense against infection at mucosal surfaces, neutralizing bacterial and viral pathogens and maintaining mucosal homeostasis [59]. IgA1 has a longer hinge region than IgA2. Changes in the O-glycosylation of the serine and threonine residues in the hinge region of IgA1 result in the formation of Gd-IgA1 [56,59]. Interestingly, heterogeneity in the O-glycoforms contained within the IgA1 hinge-region has been reported between Asian and Caucasian patients and may partially account for the increased susceptibility to IgAN in Asian populations [60]. Once Gd-IgA1 has been generated, the circulating levels have been shown to relate to worsened prognoses and outcomes in patients with IgAN [61,62].

## 4. Evidence Supporting the Involvement of APRIL in IgAN

### 4.1. Gut Mucosa–Kidney Axis in IgAN

Gd-IgA1 is thought to be produced by ASCs located in the mucosa-associated lymphoid tissue, particularly the gut-associated lymphoid tissue (GALT) [54]. IgA found in mesangial deposits in the kidneys of patients with IgAN includes the polymeric secretory form exclusively produced at the mucosal surface [56]. Moreover, although challenging to study, there is considerable evidence for a ‘gut-kidney axis’ in kidney diseases, with both the gut microbiome and diet reported to have an impact on the development and progress of IgAN [57,58,63]. An exaggerated IgA response to mucosal antigen challenge has been reported in IgAN, and it is thought that alimentary pathogens or antigens may initiate aberrant mucosal B-cell activation and Gd-IgA1 synthesis [64]. Notably, while no single pathogenic organism has been found to be associated with IgAN, it seems that a dysfunctional response to commensal organisms, driven via APRIL and/or BAFF signaling, may play a critical role [65,66].

### 4.2. APRIL Is Produced in GALT

The examination of tissue from normal human gut has shown that APRIL is expressed in GALT, lamina propria, and intestinal epithelium, with particularly strong expression in areas located close to lymphoid tissue [30]. It has been hypothesized that in healthy individuals, APRIL assists in maintaining the mucosal barrier and promotes the survival of mucosal plasma cells [30]. It is also involved in IgA class switch recombination via its binding to TACI [67]; notably, while both APRIL–TACI and BAFF–TACI binding have been shown to induce IgA class switching in vitro, only APRIL appears to have this effect in vivo [68].

### 4.3. Clinical Epidemiology

**Figure 3:**
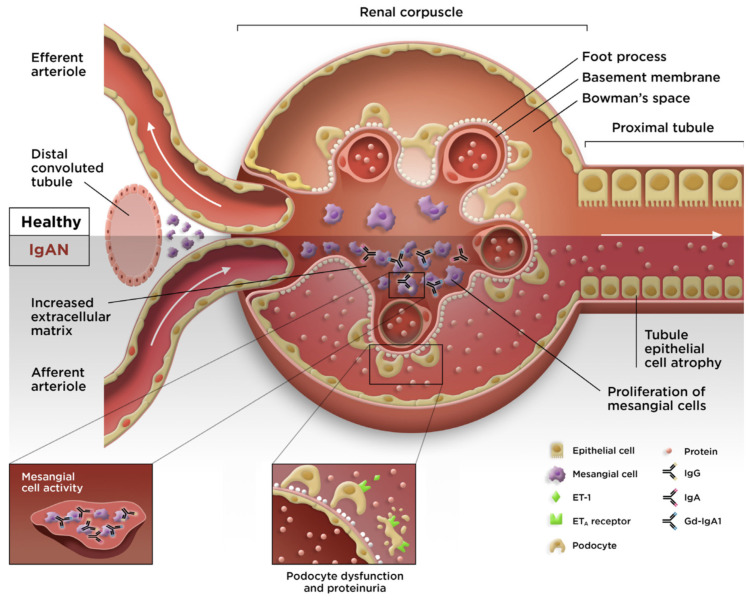
Pathogenesis of IgAN. ET_A_; endothelin A receptor; ET-1, endothelin-1; Gd-IgA1, galactose-deficient immunoglobulin A1; IgA, immunoglobulin A; IgAN, immunoglobulin A nephropathy; IgG, immunoglobulin G. Figure 3 was adapted from Lai KN, Tang SC, Schena FP, et al. IgA nephropathy. *Nat Rev Dis Primers*. **2016**, *2*, 16001, with permission from Springer Nature (https://www.springernature.com/gp accessed on 10 October 2023).

The reports in the published literature have demonstrated that the normal physiologic roles of APRIL (IgA class switching and the survival of IgA-producing plasma cells [30,67]) are implicated in the pathophysiology of IgAN, whereby gut hyperresponsiveness and elevated APRIL expression result in the increased production of Gd-IgA1, thus providing a critical link to hit 1 of the 4-hit mechanism of IgAN (Figure 3) [54]. 

Increased levels of APRIL have been observed in several clinical studies of IgAN, correlating with both Gd-IgA1 levels and disease severity (increased proteinuria and decreased estimated glomerular filtration rate [eGFR]) [56]. In a comparative study of 166 patients with IgAN and 77 healthy controls, elevated plasma levels of APRIL were observed in IgAN, accompanied by increased TACI and BCMA expression in B cells and overproduction of Gd-IgA1 [15]. In another study, B cells from patients with IgAN were found to have increased expression of Gd-IgA1 (but not total IgA) when challenged with recombinant human APRIL, while blockage of TACI and BCMA abrogated these effects [69]. Moreover, the plasma levels of APRIL in patients are associated with both the rate of eGFR loss [70] and the risk of progression to end-stage kidney disease [69]. 

#### 4.3.1. Debate 1: IgAN Pathology: APRIL, BAFF, or Both?

Data from a recent Canadian study confirmed elevated median levels of APRIL in patients with IgAN versus nonrelated household-matched control participants (1.98 ng/mL vs. 1.55 ng/mL; *p* < 0.01), and a positive correlation between serum levels of APRIL and proteinuria (Spearman’s rho = 0.28; *p* = 0.01) [71]. Notably, however, no differences in BAFF levels were observed between groups [71]. In Spanish patients with IgAN who underwent kidney transplantation, an increase in APRIL was found to precede IgAN recurrence, while the BAFF levels remained unchanged [72]. 

Conversely, other researchers, in China [73] and Italy [74] have reported elevated BAFF levels in patients with IgAN versus healthy controls. Additional, larger-scale studies are needed to determine the true picture and whether there may be demographic (e.g., race/ethnicity) or disease-related characteristics that could account for this discrepancy. 

Current drug development is split between agents that inhibit either one or both pathways. This is discussed in detail in Section 5. However, caution is required when administering agents that have a profound impact on the immune system, due to the potential for opportunistic infections [75]. This risk is likely to be higher when inhibiting both APRIL and BAFF, since altering both B-cell maturation (BAFF) and the survival of mature plasma cells (APRIL) may further escalate the safety risks. A study with a dual APRIL/BAFF inhibitor in patients with SLE reported that the incidence of treatment-emergent adverse events and infections was comparable between the two groups; however, the levels of CD19+ B cells were reduced by approximately 50% following treatment [76], which could be a safety liability for patients. Hypothetically, however, APRIL inhibition may impart less risk than dual inhibition, as APRIL inhibition does not impede B-cell maturation; thus, the pool of mature B cells and the intact T-cell responses are maintained.

#### 4.3.2. Debate 2: Does Nasopharyngeal Lymphoid Tissue Play a Major Role?

In addition to GALT, nasal-associated lymphoid tissue (NALT) in the tonsils and adenoids has also been implicated in the pathogenesis of IgAN. Plasma cells in NALT produce a greater ratio of IgA1 to IgA2 [77], and a link between oral and tonsillar microbiota and dysregulated NALT immunoreactivity has been postulated as a cause of IgAN [78,79]. Mass spectrometry data demonstrated that the IgA1 produced by ASCs in the tonsils was galactose deficient in the hinge region [80]. In a study of 24 patients with IgAN, the cells in the tonsillar germinal centers produced APRIL, indicating an upregulation of APRIL expression in this region compared with that of the control patients [81]. Moreover, this aberrant APRIL expression correlated with proteinuria [81]. 

In Japan and other Asian countries, tonsillectomy has been implemented within the treatment regimen for IgAN for the past two decades, with multiple studies reporting beneficial outcomes [82,83,84,85]. However, patients in other geographic regions have not shown the same response [86], and tonsillectomy is not routinely recommended for Caucasian patients with IgAN [87].

### 4.4. Insights from Genetic Studies

Among the GWAS conducted to search for susceptibility loci underlying IgAN, several studies have primarily served to confirm a link between race/ethnicity and disease by indicating an association with human leukocyte antigen variants [88,89]. However, a consistent association between IgAN and the chromosomal region 17p13 (encoding APRIL) has also been reported in GWAS studies, with this association remaining valid across patients with either European or East Asian ancestry [5,90]. Of note, although one GWAS suggested the involvement of BAFF in B-cell immune responses in tonsillectomy samples [91], BAFF was not identified as a locus of interest in either of the IgAN GWAS in which APRIL was detected. In another GWAS of serum protein levels in Japanese individuals, variants at the 17p13 locus of the APRIL gene and at the 17p11 locus encoding TACI were associated with levels of total protein, non-albumin protein, and immunoglobulins (including IgA) [92]. Similarly, in patients with IgAN, those with the 17p23 risk variant were found to have elevated serum IgA levels [5], further supporting the link between APRIL and IgAN pathophysiology.

### 4.5. Experimental Evidence from Animal Models

Various animal models have been used as tools to evaluate the role of APRIL in vivo [16,33,93]. These data implicated APRIL in the pathogenesis of IgAN and indicated the value of APRIL as a therapeutic target. 

## 5. Treatment of IgAN and Development of Anti-APRIL Therapies

### 5.1. Symptom Reduction and Supportive Care

Until recently, there were no disease-modifying treatments for IgAN, and the treatment guidelines were primarily focused on supportive care, with the aim of controlling blood pressure and maintaining kidney function [87]. The treatment recommendations included a renin-angiotensin system blockade using angiotensin-converting enzyme (ACE) inhibitors or angiotensin 1-receptor blockers (ARBs) [87]. Recent evidence has indicated that the sodium-glucose cotransporter-2 inhibitor dapagliflozin may be a safe and effective addition to the current standard of care [94,95]. 

For patients with continued proteinuria, short-term corticosteroid therapy may be helpful [87], but the evidence for this remains contentious [96]. In the TESTING trial, patients with high-risk IgAN received oral methylprednisolone or a placebo. While the methylprednisolone was efficacious compared to the placebo, the rates of serious adverse events (SAEs) were high (14.7% vs. 3.2%) and included two fatal infections in the methylprednisolone group. This ultimately led to the temporary discontinuation of the trial; however, the study was restarted 18 months later, as TESTING 2 [97]. In TESTING 2, a reduced steroid dose protocol was followed. The mean time-averaged proteinuria was lower in the reduced-dose methylprednisolone group than in the placebo group. The results from both studies highlighted the risks associated with steroid therapies [98].

Despite the potential safety risks associated with steroids, the first drug approved specifically for the treatment of IgAN was the corticosteroid budesonide. A delayed-release capsule formulation was approved by the US Food and Drug Administration (FDA) in 2021 for the reduction in proteinuria in adults with primary IgAN at risk of rapid disease progression, and it was conditionally approved in Europe in 2022 [99]. The formulation was designed to deliver budesonide locally in the gut, limiting systemic exposure [100], and this appears to have reduced the risk of serious infections leading to hospitalization or death [101]. Nonetheless, the remaining adverse event (AE) profile was similar to that of other systemic steroids, including a 5- to 7-fold increased risk of steroid-related events such as hirsutism, facial edema, dermatitis, acne, and hypertension when compared with a placebo [101].

The second drug approved by the FDA for the treatment of IgAN was the dual endothelin angiotensin-receptor antagonist sparsentan, which is also indicated for the reduction in proteinuria in adults with primary IgAN at risk of rapid disease progression [102]. Data from the PROTECT study indicated that after 9 months of treatment, sparsentan was able to reduce the urine protein to creatinine ratio (UPCR) to a significantly greater extent than the ARB irbesartan, although long-term data are needed to ascertain whether this translates to long-term renoprotection [103]. The overall safety profile of sparsentan was similar to that of irbesartan, although higher rates of peripheral edema, hypotension, dizziness, and anemia were observed [102,103].

### 5.2. Targeted Treatments

**Figure 4:**
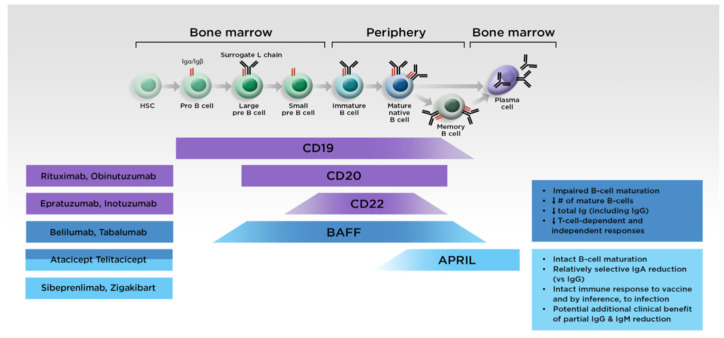
Targeted treatments for IgAN and their effects on B-cell maturation. APRIL, A PRoliferation-Inducing Ligand; BAFF, B-cell activating factor of the tumor necrosis factor family; HSC, hematopoietic stem cell; Ig, immunoglobulin.

Due to better understanding of the causes and mechanisms underlying IgAN, multiple novel therapies with various targets are now in development (Figure 4), based on the disease immunopathogenesis. However, the immunosuppressive therapies commonly used in other settings are not generally recommended for IgAN [87], and the B-cell-depleting therapy rituximab was previously shown to be ineffective [104], probably because IgA ASCs in the mucosa do not express CD20 and remain undepleted after treatment [105,106]. In addition, while a potential role for tonsillectomy, with the intention of reducing the levels of Gd-IgA1, has been described in patients from Japan [83,84,107], this is not routinely performed in other regions [86,87,108]. 

Agents that can suppress GALT immune responses, abrogate pathogenic Gd-IgA1 and autoantibody production, and thereby mitigate the resultant impacts on kidney function are particularly desirable [109]. Immunomodulators should be safer than immunosuppressants, and the fact that APRIL is expressed by mucosal epithelial cells may help create a potential focused target for the treatment of IgAN. There are currently two monoclonal antibodies targeting APRIL and three fusion proteins targeting both APRIL and BAFF via the TACI receptor under clinical investigation (Table 2).

#### 5.2.1. Anti-APRIL Monoclonal Antibodies

Sibeprenlimab (formerly VIS649) is an investigational humanized IgG2 monoclonal antibody that binds to APRIL and reduces the production of Gd-IgA1, resulting in diminished autoantibody production and decreased kidney deposition of immune complexes [110]. Data from a phase I study confirmed that sibeprenlimab was able to suppress pathogenic immunoglobulins, while preserving antibody responses to routine vaccinations [110]. Both placebo- and sibeprenlimab-treated participants had increased post-immunization titers of tetanus toxoid IgG (maximum mean increase at week 6), and the preservation of qualitative antibody responses to routine vaccine antigens was observed in the sibeprenlimab-treated group despite APRIL suppression [110]. The results from an interim analysis of the phase 2, placebo-controlled ENVISION study have also been reported [116]; compared with a placebo, intravenous sibeprenlimab administered every 4 weeks was able to reduce proteinuria at month 9 in adults with IgAN; it also produced a stable eGFR, with a favorable safety and tolerability profile. Interim biomarker analysis indicated that sibeprenlimab administration was able to suppress Gd-IgA1, IgA, IgM, and, to a lesser extent, IgG [117]. Moreover, the preliminary data showed that responses to mRNA-based vaccines against coronavirus disease 2019 were found to be intact in sibeprenlimab recipients, with vaccinated participants achieving protective levels of IgG that were specific to the receptor-binding domain of the severe acute respiratory syndrome coronavirus 2 [118]. The induced antibody half-life and the time-above-protective threshold were both comparable between the sibeprenlimab and placebo recipients. Based on these results, the phase III VISIONARY study (NCT05248646; N = 470 participants) of sibeprenlimab administered subcutaneously every 4 weeks was initiated in March 2022. A long-term phase II/III open-label extension study (NCT05248659) was also initiated.

Zigakibart (BION-1301), a humanized IgG4 anti-APRIL monoclonal antibody, was evaluated in an open-label phase I study in healthy volunteers and was reported to be well tolerated, while providing dose-dependent and durable reductions in serum levels of free APRIL, IgA and Gd-IgA1, and IgM [111]. Although a phase I/II study in multiple myeloma (NCT03340883) was terminated early due to a lack of response, the interim results from another small phase I/II open-label study in patients with IgAN demonstrated that zigakibart administered every 2 weeks was well tolerated for at least 12 weeks and produced reductions in serum levels of immunoglobulins, free APRIL, and proteinuria [111]. A phase III study in adults with IgAN (NCT05852938) is currently enrolling.

#### 5.2.2. Fusion Protein Antagonists Targeting Both APRIL and BAFF

Atacicept, a fusion protein comprising recombinant TACI and human IgG1, was originally developed for the treatment of immune-mediated disorders, including SLE and multiple sclerosis [119]. However, this is no longer being pursued [119,120,121]. Development in other indications continued, and the results of the phase II JANUS study in patients with IgAN reported a dose-dependent decrease in serum IgA, IgG, IgM, and Gd-IgA1, with an acceptable safety profile after 72 weeks of atacicept treatment [112]. Topline results from the phase II ORIGIN study (NCT04716231) in IgAN were recently released, indicating that atacicept met its primary endpoint of change from the baseline in UPCR after 24 weeks when compared with a placebo, and it was generally well tolerated with no increased rate of infections [113]. Based on these results, phase III development in IgAN is now underway.

Telitacicept, another fusion protein (recombinant TACI receptor linked to the Fc domain of human IgG), is currently approved in China for the treatment of SLE [122], and clinical development in other indications is ongoing. In a phase II study in Chinese patients with IgAN, telitacicept reduced proteinuria and stabilized eGFR after 6 months of treatment when compared with a placebo, and the treatment-emergent adverse events were mild or moderate with no severe adverse events reported [114]. A second phase II study is currently recruiting in the USA (NCT04905212). In addition, a phase III study in patients with primary IgAN (NCT05799287) and a phase II/III study in patients with refractory IgAN (NCT05596708) are planned. However, data derived from an SLE study in which a reduction of approximately 50% was observed in levels of CD19+ B cells following telitacicept treatment [76] suggests that careful monitoring of patients may be necessary to avoid the risk of serious infection.

The fusion protein povetacicept (ALPN-303; TACI-Ig) was developed as a high-affinity APRIL/BAFF antagonist [123]. The preclinical data indicated enhanced target binding affinity and greater inhibitory activity when compared with atacicept and telitacicept [123], and povetacicept was found to be well tolerated in healthy volunteers [115]. Povetacicept recently entered phase I clinical development (RUBY-3; NCT05732402) in patients with autoimmune kidney diseases, including IgAN. 

## 6. Future Directions

Although studies have implicated APRIL in the pathogenesis of IgAN, more evidence to clarify and confirm the direct role of APRIL in the development and the progression of this condition is needed. Several aspects of anti-APRIL targeting also require further investigation. Chief among these are the questions of whether single (APRIL) or dual (APRIL/BAFF) inhibition would be well tolerated, the most effective and safe in patients with IgAN, and whether there would be class-related differences in the safety profiles. While agents of both types have entered clinical development, the data in IgAN patients remain limited; large-scale phase III studies and head-to-head evaluations are needed to address these points more fully. 

Currently, the efficacy data for both anti-APRIL monoclonal antibodies and dual APRIL/BAFF fusion protein antagonists appear positive [111,113,114,116]. Thus far, the safety signals have also been reassuring. In interim analyses of phase II studies, sibeprenlimab was not associated with any drug-related SAEs [116]; similarly, in a smaller study population, zigakibart did not induce any SAEs [111]. Atacicept-treated patients in the phase II JANUS study did not report any SAEs that were judged to be treatment-related [112]. In the phase II study in China, 1 out of 30 telitacicept-treated patients reported a treatment-related SAE, specifically a severe injection-site reaction [114]. 

Due to the increased risk of opportunistic infection when targeting the immune system [94], studies must continue to monitor for relevant AEs during ongoing development in IgAN. The use of steroids in the TESTING trial was found to increase the risk of infection, and it led to fatal AEs in two patients [97]. Thus, an immunomodulatory drug that has a minimal impact on the immune response is needed. To date, there have been no indications that any of the targeted agents discussed may increase the risk of severe or fatal infection in IgAN patients, although the results of large-scale studies and longer-term data are awaited to confirm this. However, compared with the agents targeting BAFF, anti-APRIL therapies may have a hypothetical advantage over dual APRIL/BAFF fusion proteins since the inhibition of APRIL should not affect the B-cell development and maturation processes. Phase III studies in larger patient populations may provide further corroborative clinical evidence.

## 7. Conclusions

The cytokine APRIL plays an important role in the pathogenesis of IgAN. Elevated circulating APRIL levels found in patients with IgAN correlate with disease severity. Experimental models of IgAN have also shown that APRIL inhibition reduces disease severity and slows progression. Moreover, the preliminary data have shown promising efficacy with an acceptable safety profile. As a result, there are multiple clinical trials evaluating the role of anti-APRIL therapies in IgAN. The results of the large-scale phase III clinical studies are eagerly awaited.

## Figures and Tables

**Table 1:** Overview of BAFF and APRIL functions by receptor type.

Function	APRIL	BAFF	Receptors Mediating These Functions			
			TACI	BCMA	BAFF-R	Proteoglycans
B-cell survival and maturation		X	X	X	X	
Plasma blast and plasma cell differentiation and survival	X			X		
Maintenance of B1 cell	X					X
T-cell-independent antibody response	X	X	X	X	X	
Antibody class switch and recombination	X		X			
Plasma cell trafficking	X					X

X; data are available to support this function. [4,13,22,23] APRIL, A PRoliferation-Inducing Ligand; BAFF, B-cell activating factor of the tumor necrosis factor family; BAFF-R, BAFF receptor; BCMA, B-cell maturation antigen; TACI, transmembrane activator calcium modulator and cyclophilin ligand interactor.

**Table 2:** Anti-APRIL therapeutic candidates for IgAN.

Potential Treatment (Sponsor)	Drug Type	IgAN Clinical Trials	Status
Sibeprenlimab (Otsuka Pharmaceutical Co., Ltd./Visterra Inc.)	Anti-APRIL monoclonal antibody	NCT03719443	Phase I complete [110]
		NCT04287985	Phase II complete
		NCT05248646	Phase III recruiting
		NCT05248659	Phase II/III enrolling
Zigakibart (Chinook Therapeutics, Inc./Novartis)	Anti-APRIL monoclonal antibody	NCT05508204	Phase I complete [111]
		NCT03945318/NCT04684745	Phase I/II ongoing [111]
		NCT05852938	Phase III enrolling
Atacicept (Vera Therapeutics/Merck KGaA)	TACI-IgG fusion protein	NCT02808429	Phase II complete [112]
		NCT04716231	Phase II ongoing [113] Phase III recruiting
Telitacicept (Yantai Rongchang Pharmaceuticals, Ltd./RemeGen Co., Ltd.)	TACI receptor-IgG fusion protein	NCT04291781	Phase II complete [114]
		NCT04905212	Phase II recruiting
		NCT05596708	Phase II/III planned
		NCT05799287	Phase III planned
Povetacicept (Alpine Immune Sciences, Inc.)	TACI-Ig fusion protein	NCT05034484	Phase I complete [115]
		NCT05732402	Phase I recruiting

APRIL, A PRoliferation-Inducing Ligand; Ig, immunoglobulin; IgAN, immunoglobulin A nephropathy; TACI, transmembrane activator calcium modulator and cyclophilin ligand (CAML) interactor.

## Data Availability

No new data were created or analyzed in this study. Data sharing is not applicable to this article.
